# In Situ Formation of AgCo Stabilized on Graphitic Carbon Nitride and Concomitant Hydrolysis of Ammonia Borane to Hydrogen

**DOI:** 10.3390/nano8050280

**Published:** 2018-04-26

**Authors:** Qi Wang, Caili Xu, Mei Ming, Yingchun Yang, Bin Xu, Yi Wang, Yun Zhang, Jie Wu, Guangyin Fan

**Affiliations:** 1 College of Chemistry and Materials Science, Sichuan Normal University, Chengdu 610068, China; wangqi@sicnu.edu.cn (Q.W.); cailixu@sicnu.edu.cn (C.X.); meiming@sicnu.edu.cn (M.M.); zhangyun@sicnu.edu.cn (Y.Z.); wujie@sicnu.edu.cn (J.W.); 2 College of Resources and Environment, Chengdu University of Information Technology, Chengdu 610225, China; yangyingchun@cuit.edu.cn; 3 School of Chemical and Environmental Engineering, Sichuan University of Science & Engineering, Zigong 643000, China; jwdxb@suse.edu.cn

**Keywords:** graphitic carbon nitride, bimetallic catalyst, ammonia borane, hydrogen generation

## Abstract

The development of highly-efficient heterogeneous supported catalysts for catalytic hydrolysis of ammonia borane to yield hydrogen is of significant importance considering the versatile usages of hydrogen. Herein, we reported the in situ synthesis of AgCo bimetallic nanoparticles supported on g-C_3_N_4_ and concomitant hydrolysis of ammonia borane for hydrogen evolution at room temperature. The as-synthesized Ag_0.1_Co_0.9_/g-C_3_N_4_ catalysts displayed the highest turnover frequency (TOF) value of 249.02 mol H_2_·(mol_Ag_·min)^−1^ for hydrogen evolution from the hydrolysis of ammonia borane, which was higher than many other reported values. Furthermore, the Ag_0.1_Co_0.9_/g-C_3_N_4_ catalyst could be recycled during five consecutive runs. The study proves that Ag_0.1_Co_0.9_/g-C_3_N_4_ is a potential catalytic material toward the hydrolysis of ammonia borane for hydrogen production.

## 1. Introduction

The widespread applications of fossil fuels result in the resource exhaustion and serious environmental pollution. Hydrogen has attracted increasing attention as a clean and renewable energy carrier [1]. However, the difficulties in controllable storage and safe delivery of hydrogen restrict its applications entirely. Chemical hydrides are widely investigated as raw materials to produce hydrogen [2,3,4,5,6,7,8], among which ammonia borane (AB) has been proven to be a promising hydrogen storage material to generate hydrogen. Hydrogen can be stoichiometrically produced with appropriate catalysts.

For the hydrolysis of AB, noble metals, such as Pt [9,10], Rh [11], Ru [5,7,12,13,14,15], and Pd [15,16], are intensively studied as the catalytic active sites, while the less expensive metals, such as Au and Ag, are rarely explored because of their low catalytic activities. To promote their catalytic performances, and simultaneously reduce the usage of these metals, bimetallic catalysts with earth-abundant and low-cost metals have aroused much attention because of their enhanced catalytic activity toward the hydrolysis of AB in aqueous solution. Various supports, including graphene and metal-organic-frameworks, are explored to prepare supported catalysts for the hydrolysis of AB. For example, Ag@Co/graphene was prepared and selected as the catalyst for the hydrolysis of AB with a turnover frequency (TOF) of 102.4 mol H_2_·(mol_Ag_·min)^−1^ [17]. The AuCo@MIL-101 [18] catalyst reported by Xu et al. exhibited a TOF value of 23.5 mol H_2_·(mol_Au_·min)^−1^. The Au-Co@CN nanoparticles prepared by Guo et al. displayed a TOF of 48.3 mol H_2_·(mol_Au_·min)^−1^ [19]. Despite these advances, the catalytic performance of the Ag- and Au-based catalysts are still not satisfactory enough and developing catalysts with high efficiencies is still challenging.

As is well-known, the catalytic property of a heterogeneous catalyst is closely correlated to the nature of the catalyst support [20]. Synthesis of catalyst supports in a convenient and inexpensive way is very attractive for practical applications. Graphitic-carbon nitride (g-C_3_N_4_) synthesized through the pyrolysis of inexpensive urea is such a supporting material with superior chemical and physical properties [21], which can provide more active sites for reaction [22]. Therefore, g-C_3_N_4_ is widespreadly applied as electrochemical catalysts [21,23] and photocatalysts [24,25]. Herein, we reported the in situ formation of an AgCo bimetallic catalyst and concomitant hydrolysis of AB to produce hydrogen at room temperature (25 °C). The AgCo bimetallic catalyst displayed an outstanding performance toward the catalytic hydrolysis of AB. A very high TOF of 249.02 mol H_2_·(mol_Ag_·min)^−1^ was observed because of the synergistic effect of Ag and Co metals, as well as the interaction between metal and support. The present work may offer new opportunity for design and synthesis of highly-active catalysts with g-C_3_N_4_ as the catalyst support for various catalytic applications.

## 2. Experimental

### 2.1. Materials and Methods

CoCl_2_∙6H_2_O (AR) was purchased from Aladdin Industrial Inc. (Shanghai, China). AgNO_3_ (AR) was provided by Tianjin North Industrial (Tianjin, China). AB complex (90%) and urea were bought form Sigma-Aldrich (St. Louis, MO, USA). All the chemicals were used without further purification. Ultrapure water was used in all tests. 

The morphology and particle sizes of Ag_0.1_Co_0.9_/g-C_3_N_4_ and recycled Ag_0.1_Co_0.9_/g-C_3_N_4_ were measured by Transmission electron microscopy (TEM) with FEI Tecnai G20 (Hillsboro, OR, USA) operating at 200 kV. Powder X-ray diffraction (XRD) patterns were recorded by a Panalytical X'Pert PRO X-ray diffractometer (Egham, Surrey, UK) with Cu Kα radiation operated at 40 kV and 40 mA. X-ray photoelectron spectroscopy (XPS) spectra were collected on a Thermo ESCALAB 250 Axis Ultra spectrometer (Waltham, MA, USA) with a monochromatic Al Kα source (*hυ* = 1486.6 eV). Fourier transform infrared (FTIR) spectra (BRUKER VERTEX70, GER) in the 400-4000 cm^−1^ region were obtained on a Thermo Nicolet 870 instrument. Inductively coupled plasma-optical emission spectrometry (ICP-OES) analysis was carried out on SPECTRO ARCOS spectrometer (SPECTRO, Kleve, Germany).

### 2.2. Preparation of Ag_x_Co_1−x_/g-C_3_N_4_

g-C_3_N_4_ was synthesized via the pyrolysis method by using urea as the raw material. Specifically, urea was deposited in a porcelain crucible and heated at 300 °C for 1 h, and followed by calcining at 550 °C for another 3 h. After cooling to room temperature, 10 mg of the as-prepared g-C_3_N_4_ sample was transferred into a 25 mL flask containing a certain amount of water. Then the desired amount of AgNO_3_ aqueous solution (5.50 mg/mL) and CoCl_2_ solution (8.67 mg/mL) were added into the flask. The total volume of the solution was controlled at 4.0 mL. Finally, the mixture was ultrasonicated for 20 min to get a uniform suspension. The reaction temperature was controlled at 25 °C by a water bath. AB aqueous solution (1.0 mmol AB was dissolved in 1.0 mL H_2_O) was rapidly injected into the reactor. During the reaction process, the volume of H_2_ was measured by the water-displacement method. For comparison, the molar ratio of Ag/Co was controlled at 1:9, 2:8, 3:7, 7:3, 8:2, 9:1, 10:0, and 0:10. The Ag and Co loadings determined by ICP-OES are shown in Table S1 in detail.

For comparison, Al_2_O_3_ and Mobil Composition of Matter N. 41 (MCM-41) were also used as supports for depositing Ag_0.1_Co_0.9_ bimetallic catalysts under the same procedures. For stability tests, the catalyst was recovered from the reaction mixture and washed with water three times. Then, the catalyst was applied for the next run by adding another equivalent of AB solution into the mixture. The catalytic recycling was repeated five times.

## 3. Results and Discussion

**Figure 1:**
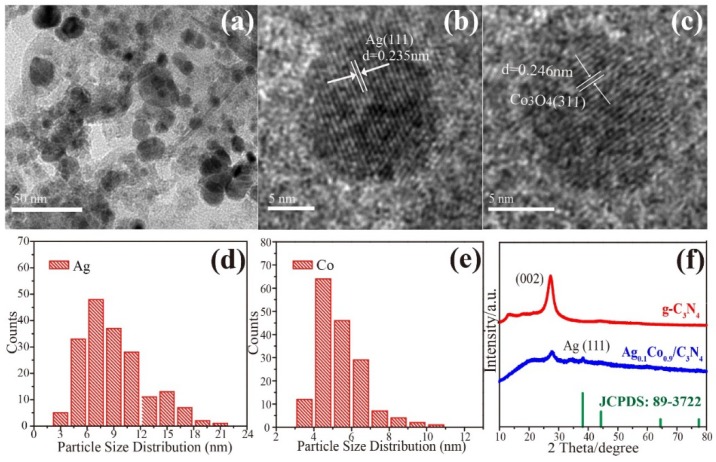
(**a**) TEM images of Ag_0.1_Co_0.9_/g-C_3_N_4_. HRTEM images of (**b**) Ag nanoparticles and (**c**) Co_3_O_4_ nanoparticles. (**d**) Particle size distribution of Ag nanoparticles. (**e**) Particle size distribution of Co nanoparticles. (**f**) XRD patterns of g-C_3_N_4_ and Ag_0.1_Co_0.9_/g-C_3_N_4_.

The morphology of the as-synthesized Ag_0.1_Co_0.9_/g-C_3_N_4_ sample was detected by TEM, and the results are shown in Figure 1. It can be seen that the particles are homogenously anchored on the support (Figure 1a). High-resolution TEM images were further recorded to confirm their syntheses. As shown in Figure 1b, the lattice spacing of 0.235 nm is ascribed to the Ag (111) plane [17], whereas the d-spacing of 0.246 nm is correlated to the Co_3_O_4_ (311) plane (Figure 1c). The presence of oxidized cobalt species could be assigned to the oxidation of metallic cobalt in the sample preparation. The mean particle size of the Ag and Co nanoparticles are calculated as shown in Figure 1d,e. The average sizes of Ag and Co_3_O_4_ nanoparticles are calculated to be 9.04 nm and 5.37 nm, respectively. The results show that Ag and Co are successfully supported on the g-C_3_N_4_ support. It should be pointed out that no alloy and core-shell structure of Ag and Co bimetallic nanoparticles are observed in the present synthetic conditions. Figure 1f illustrates the XRD patterns of the as-synthesized samples. The diffraction peak located at 2θ = 27.4° is attributed to (002) crystal plane of g-C_3_N_4_, which is assigned to the characteristic inter-planar stacking conjugated aromatic structure after the polymerization of urea [26]. In the XRD patterns of Ag_0.1_Co_0.9_/g-C_3_N_4_, the diffraction peak detected at 2θ = 38.26° is assigned to the (111) crystal plane of Ag (JCPDS No. 89-3722). Nevertheless, no obvious characteristic peaks of cobalt species are detected.

**Figure 2:**
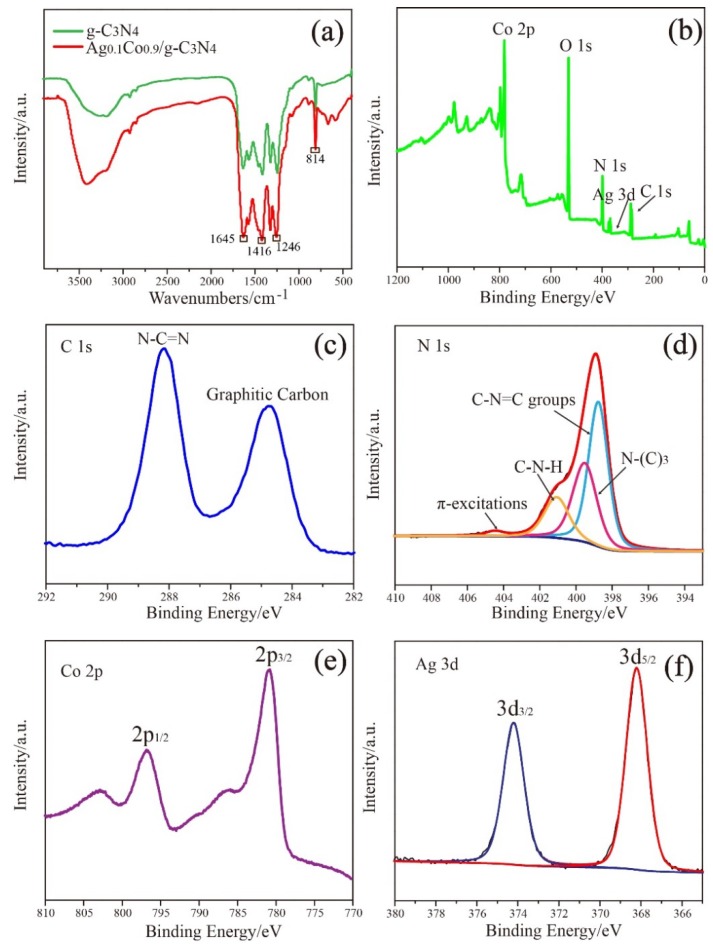
(**a**) FTIR spectra of g-C_3_N_4_ and Ag_0.1_Co_0.9_/g-C_3_N_4_. (**b**) XPS of the survey scan of the catalyst. XPS spectra of (**c**) C 1s, (**d**) N 1s, (**e**) Co 2p, and (**f**) Ag 3d.

FTIR spectra was further applied to analyze the structure of the g-C_3_N_4_ support. As displayed in Figure 2a, a broad band situated at 3200–3500 cm^−1^ is detected, which is assigned to the stretching vibration modes of N–H bonds and the surface absorbed oxygen-containing groups, such as hydroxyl groups. The observation of the N–H stretching vibration modes verifies that the carbon nitride layer contained some uncondensed amine functional groups. Several strong bands appeared at 1246 cm^−1^, 1416 cm^−1^, and 1645 cm^−1^ correspond to typical stretching modes of the carbon-nitrogen (C-N) heterocycle. The characteristic breathing modes of the triazine units at 814 cm^−1^ [27] indicate the successful synthesis of g-C_3_N_4_ support. In addition, no apparent changes between the g-C_3_N_4_ and Ag_0.1_Co_0.9_/g-C_3_N_4_ spectra are detected, illustrating that the stabilization of Ag and Co do not affect the structure of the support.

XPS characterization was employed to investigate the electronic states of the elements in Ag_0.1_Co_0.9_/g-C_3_N_4_. Figure 2b displays the XPS survey scan of the catalyst, in which carbon, nitrogen, oxygen, silver, and cobalt elements are detected, implying the successful synthesis. According to the high-resolution XPS spectrum of C1s (Figure 2c), the presence of the N–C=N groups, as well as graphitic carbon, are verified because of the appearance of two characteristic peaks at 288.2 eV and 284.9 eV [28,29]. With regard to the N1s spectrum (Figure 2d), the presence of C–N–C groups, the three N-bonded C atoms of aromatic cycles, and the π-excitations can be confirmed through the observation of characteristic peaks with binding energies of 398.8 eV, 400.9 eV, and 404.5 eV, respectively. The presence of N–(C)_3_ or H–N–(C)_2_ groups are verified according to the XPS peak with a binding energy of 399.5 eV. [29,30]. As shown in Figure 2e, the Co 2p core-level XPS spectrum can be divided into two peaks with binding energies of 780.8 eV and 796.8 eV, indicating the formation of Co_3_O_4_. The presence of oxidized cobalt is probably attributed to the oxidation of Co during the catalyst preparation since metallic cobalt is sensitive to air [5,31,32]. From the Ag 3d spectrum in Figure 2f, it can be seen that the Ag species are in the metallic states, since two distinct peaks with binding energies of 374.2 and 368.2 eV are observed [17,33].

**Figure 3:**
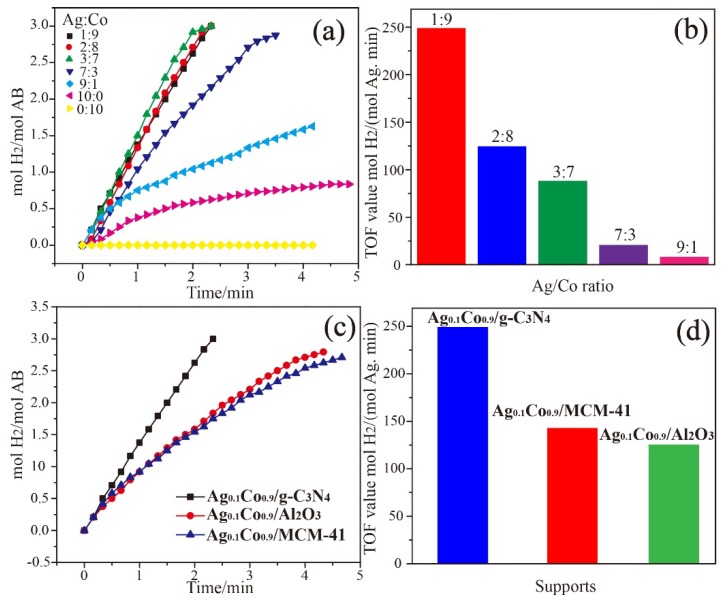
(**a**) The plots of mol H_2_/mol AB versus time for different ratios of Ag/Co. (**b**) TOF values of different ratios of Ag/Co. (**c**) The plots of mol H_2_/mol AB versus time graph for different catalysts. (**d**) TOF values of different catalysts toward the hydrolysis of AB.

**Figure 4:**
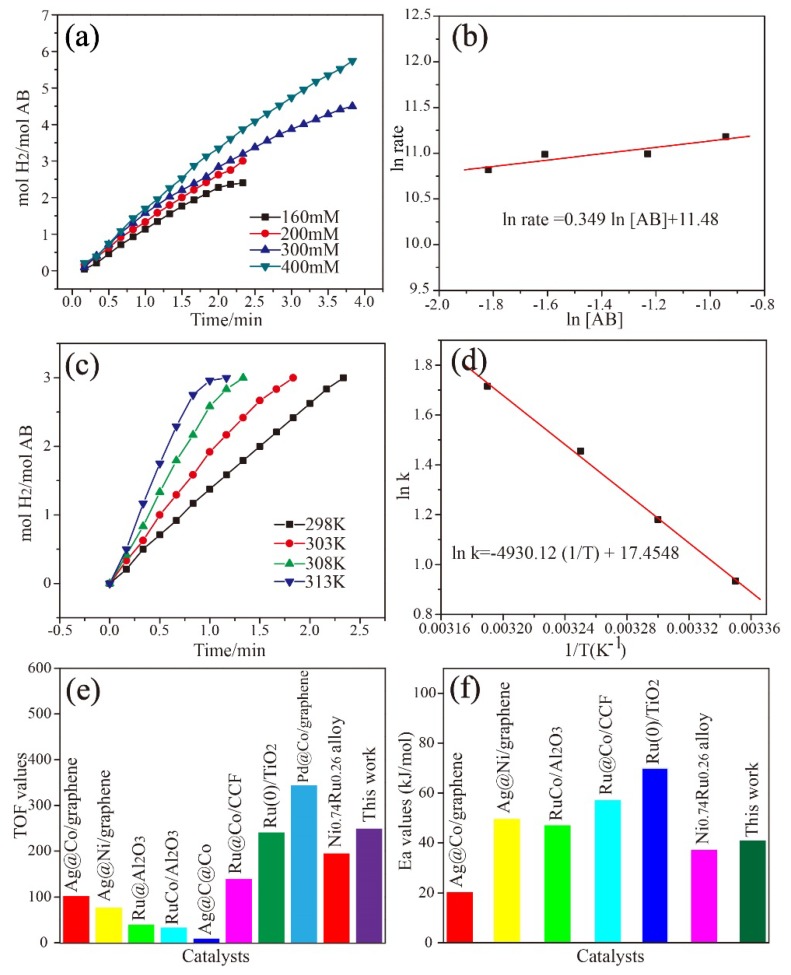
(**a**) The plots of mol H_2_/mol AB versus reaction time at different AB concentrations. (**b**) Plot of ln (rate) versus ln [AB]. (**c**) The plots of mol H_2_/mol AB versus time for different temperatures. (**d**) The Arrhenius plot for the hydrogen release of AB. (**e**,**f**) Comparison of TOF values and E_a_ values among this work and the reported literature.

Figure 3a shows the effect of the Ag/Co molar ratios in Ag_x_Co_1−x_/g-C_3_N_4_ on the hydrolysis of AB. It is observed that Ag_0.1_Co_0.9_/g-C_3_N_4_, Ag_0.2_Co_0.8_/g-C_3_N_4_, and Ag_0.3_Co_0.7_/g-C_3_N_4_ catalysts show higher catalytic activities than other investigated catalysts, whereas the hydrolysis reaction is incomplete over the Ag_1.0_Co_0_/g-C_3_N_4_, Ag_0_Co_1.0_/g-C_3_N_4_, Ag_0.7_Co_0.3_/g-C_3_N_4_, and Ag_0.9_Co_0.1_/g-C_3_N_4_ catalysts. Especially, the highest TOF of 249.09 mol H_2_·(mol_Ag_·min)^−1^ is observed with the Ag_0.1_Co_0.9_/g-C_3_N_4_ catalyst, indicating its high catalytic activity toward the hydrolysis of AB. To obtain detailed information on their catalytic performances, the XRD patterns of the investigated samples were recorded, and the results are shown in Figure S1. From the XRD patterns, it can be seen that the strong and sharp diffraction peaks of Ag are detected with the decrease of Co content, indicating that the generation of large Ag nanoparticles. The broad and weak characteristic peaks of Ag observed in Ag_0.1_Co_0.9_/g-C_3_N_4_ indicate the formation of smaller Ag nanoparticles with higher Co contents. The results verify that the addition of more Co species is benefited to the control of Ag particle size in the synthesized samples. It is well-established that the small-sized metal nanoparticles can provide more highly surface-active sites for catalytic applications. Therefore, the presence of small Ag nanoparticles in Ag_0.1_Co_0.9_/g-C_3_N_4_ are responsible for the high catalytic activity. In addition, the Ag_0.1_Co_0.9_/g-C_3_N_4_ catalyst also exhibits much higher catalytic activity compared with the reported results, such as Ag@Co/graphene (102.4 mol H_2_·(mol_Ag_·min)^−1^) [17], Ag@Ni/graphene (77 mol H_2_·(mol_Ag_·min)^−1^) [17], Ru@Al_2_O_3_ (39.6 mol H_2_·(mol_Ru_·min)^−1^) [34], Ag@C@Co (8.93 mol H_2_·(mol_Ag_·min)^−1^) [33], RuCo@Al_2_O_3_ (32.9 mol H_2_·(mol_Ru_·min)^−1^) [14], Pd@Co/graphene (408.9 mol H_2_·(mol_Pd_·min)^−1^) [16], and Ni_0.74_Ru_0.26_ alloy (194.8 mol H_2_·(mol_Ru_·min)^−1^) [13] (Figure 4e).

We also prepared the MCM-41 and Al_2_O_3_ supported Ag_0.1_Co_0.9_ bimetallic nanoparticles and investigated their catalytic activity toward the hydrolysis of AB. From Figure 3c, we can see that the Ag_0.1_Co_0.9_/g-C_3_N_4_ can quickly catalyze the complete hydrolysis of AB in less than 2.5 min. However, Ag_0.1_Co_0.9_/MCM-41 and Ag_0.1_Co_0.9_/Al_2_O_3_ require longer time to complete the hydrolysis reaction. As exhibited in Figure 3d, Ag_0.1_Co_0.9_/g-C_3_N_4_ has a much higher catalytic activity with a TOF of 249.09 mol H_2_·(mol_Ag_·min)^−1^, while the TOF values of Ag_0.1_Co_0.9_/MCM-41 and Ag_0.1_Co_0.9_/Al_2_O_3_ are 143.1 and 124.81 mol H_2_·(mol_Ag_·min)^−1^, respectively. These results confirm that the superior catalytic performance of Ag_0.1_Co_0.9_/g-C_3_N_4_ catalyst is correlated with the nature of the g-C_3_N_4_ support.

Kinetic studies of AB hydrolysis over Ag_0.1_Co_0.9_/g-C_3_N_4_ catalyst were performed at different substrate concentrations and reaction temperatures. Figure 4a displays the effect of AB concentration on the catalytic hydrolysis of AB over the Ag_0.1_Co_0.9_/g-C_3_N_4_ catalyst. The reaction rate of the initial hydrogen evolution is acquired by fitting the linear part of each plot. Figure 4b shows the plots of ln (rate) vs. ln(AB). The fitting line with a slope of 0.349 indicates that the hydrolysis mechanism for the hydrolysis of AB with AgCo/g-C_3_N_4_ catalyst is differentiated with the literature [5,10]. We further performed the hydrolysis of AB under varied reaction temperatures to calculate the activation energy (E_a_) value of the hydrolysis reaction over Ag_0.1_Co_0.9_/g-C_3_N_4_. Notably, the reaction temperature possesses a profound effect on the hydrolysis reaction. As illustrated in Figure 4c, the time of completion of the reaction toward hydrogen evolution from the hydrolysis of AB decreases with the increase of the reaction temperature. Consequently, the E_a_ value of the catalytic hydrolysis of AB over Ag_0.1_Co_0.9_/g-C_3_N_4_ catalyst is 40.91 kJ/mol, calculated by fitting the Arrhenius plot in Figure 4d. This value was smaller than most of the reported literature as listed in Figure 4f, such as Ru@Al_2_O_3_ (48 kJ/mol) [34], RuCo@Al_2_O_3_ (47 kJ/mol) [14], Ru@Co/CCF (57.02 kJ/mol) [35], and Ru(0)/TiO_2_ (70 kJ/mol) [12], but higher than Ag@Co/graphene (20.3 kJ/mol) [17] and Ni_0.74_Ru_0.26_ alloy (37.18 kJ/mol) [13]. The lower activation energy indicates the favorable reaction kinetic of AB hydrolysis catalyzed by the Ag_0.1_Co_0.9_/g-C_3_N_4_ catalyst.

**Figure 5:**
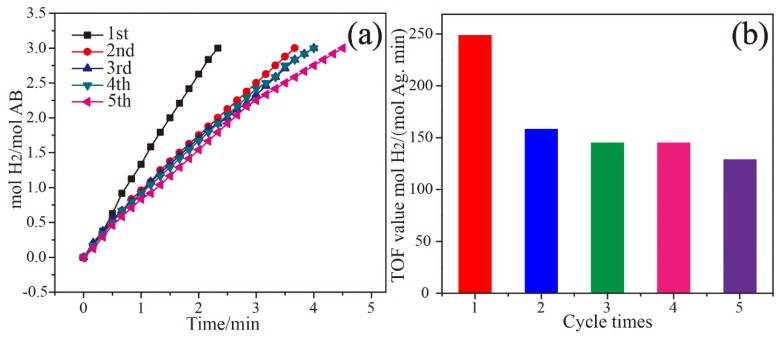
(**a**) The plots of mol H_2_/mol AB versus time during the catalyst stability tests. (**b**) The TOF values for AB hydrolysis over Ag_0.1_Co_0.9_/g-C_3_N_4_ after each cycle.

Figure 5a shows the stability and reusability of the catalyst. Notably, the catalyst still can release three equivalent H_2_ per mole AB after recycled for five times, indicating the good recyclability of Ag_0.1_Co_0.9_/g-C_3_N_4_. However, the catalyst undergoes a slight loss of activity with the increase of recycling tests. The TOF value of the fifth run is 129.16 mol H_2_·(mol_Ag_·min)^−1^, preserving 52% of the initial value. To obtain more information on the catalytic activity loss, the liquid of the reaction mixture after each run was separated for the catalytic test. The results indicate that no hydrogen evolution is observed. Therefore, TEM was performed to analyze the structure and morphology of the recycled Ag_0.1_Co_0.9_/g-C_3_N_4_ catalyst. It is observed that Ag and Co nanoparticles on the g-C_3_N_4_ surface maintain their morphologies after recycling (Figure S2a) with the mean size of 9.45 nm and 5.60 nm (Figure S2b,c), indicating that the deactivation of the catalyst is presumably ascribed to the slight increase of the particle size during the recycling and the probable increase of the viscosity of the reaction solution and/or the increasing metaborate concentration during AB hydrolysis.

## 4. Conclusions

In summary, the AgCo/g-C_3_N_4_ nanoparticles were prepared via the co-reduction of the aqueous solution of silver nitrate and cobalt chloride. The bimetallic catalyst showed synergistic effect and excellent catalytic properties for the hydrolysis of AB to produce hydrogen at 25 °C. The Ag_0.1_Co_0.9_/g-C_3_N_4_ showed highest activity with a TOF of 249.02 mol H_2_·(mol_Ag_·min)^−1^ and a lower E_a_ of 40.91 kJ/mol. The good stability and reusability indicate that the present catalyst is a promising material for the hydrolysis of AB to yield hydrogen.

## Supplementary Materials

The following are available online at http://www.mdpi.com/2079-4991/8/5/280/s1. Figure S1: the XRD patterns of the g-C_3_N_4_ and Ag_X_Co_1−X_/g-C_3_N_4_, Figure S2: (a) the TEM image of Ag_0.1_Co_0.9_/g-C_3_N_4_ after five recycling runs. (b) Particle size distribution of Ag nanoparticles after 5 recycling runs. (c) Particle size distribution of Co nanoparticles after 5 recycling runs, Table S1: Ag and Co loadings determined by ICP-OES.
