# Supplementary material for: In Situ Formation of AgCo Stabilized on Graphitic Carbon Nitride and Concomitant Hydrolysis of Ammonia Borane to Hydrogen
Source: Nanomaterials (Basel). 2018 Apr 26;8(5):280. doi: 10.3390/nano8050280 (PMC5977294; doi:10.3390/nano8050280)
Supplement: Supplementary file 1 [file nanomaterials-08-00280-s001.pdf]

In Situ Formation of AgCo Stabilized on Graphitic Carbon Nitride and Concomitant Hydrolysis of Ammonia Borane to Hydrogen

Qi Wang [1], Caili Xu [1], Mei Ming [1], Yingchun Yang [2], Bin Xu [3], Yi Wang [1,*], Yun Zhang [1], Jie Wu [1] and Guangyin Fan [1,*]

[1] College of Chemistry and Materials Science, Sichuan Normal University, Chengdu 610068, China; wangqi@sicnu.edu.cn (Q.W.); cailixu@sicnu.edu.cn (C.X.); meiming@sicnu.edu.cn (M.M.); zhangyun@sicnu.edu.cn (Y.Z.); wujie@sicnu.edu.cn (J.W.)

[2] College of resources and environment, Chengdu University of Information Technology, Chengdu 610225, China; yangyingchun@cuit.edu.cn

[3] School of Chemical and Environmental Engineering, Sichuan University of Science & Engineering, Zigong 643000, China; jwdxb@suse.edu.cn

* Correspondence: yiwang@sicnu.edu.cn (Y.W.); fanguangyin@sicnu.edu.cn (G.F.); Tel.: +86-28-8476-0802 (G.F.)

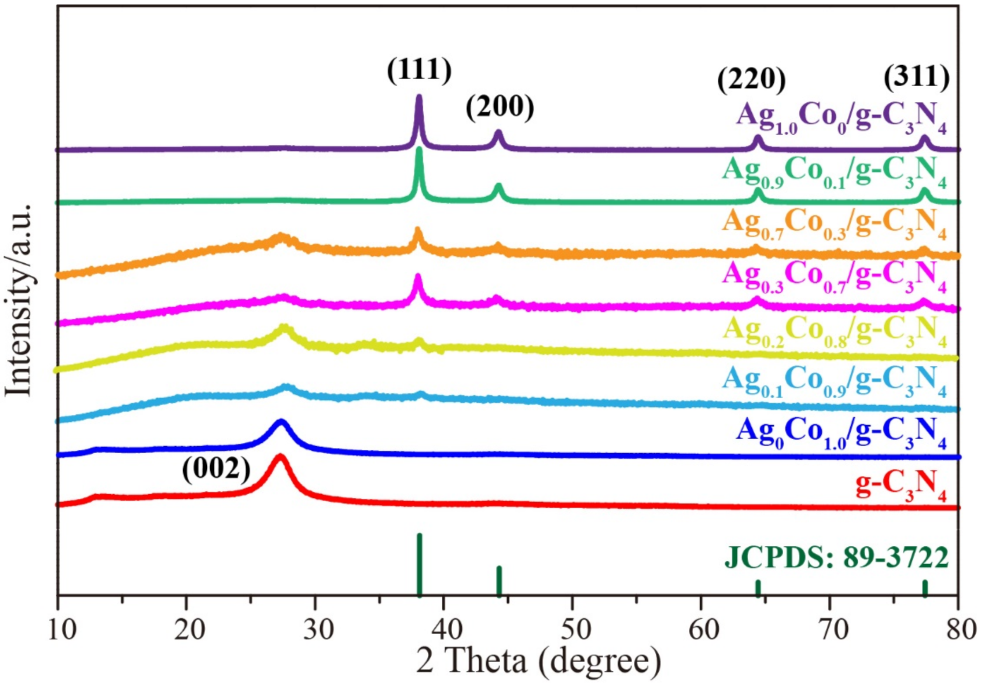

Figure S1. The XRD patterns of the g-C_3N_4 and Ag_xCo_{1-x}/g-C_3N_4.

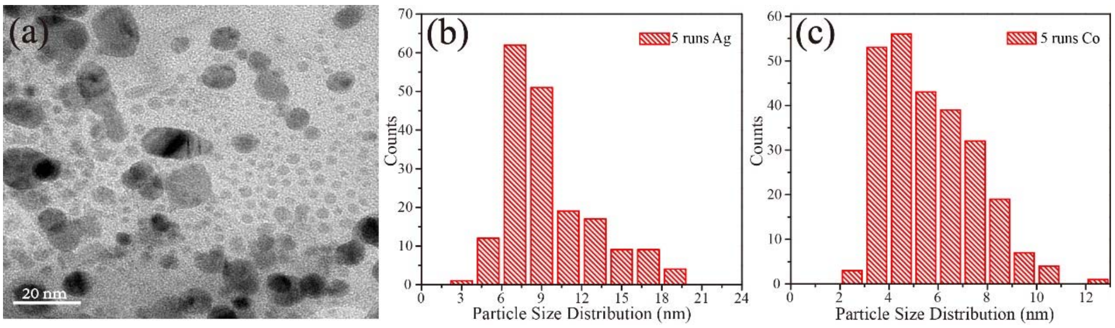

Figure S2. (**a**) the TEM image of $Ag_{0.1}Co_{0.9}$/g-C_3N_4 after five recycling runs. (**b**) Particle size distribution of Ag nanoparticles after 5 recycling runs. (**c**) Particle size distribution of Co nanoparticles after 5 recycling runs.

Table S1. Ag and Co loadings determined by ICP-OES.

Catalyst	Ag loading	Co loading
$Ag_0Co_{1.0}/g\text{-}C_3N_4$	0	21.0%
$Ag_{0.1}Co_{0.9}/g\text{-}C_3N_4$	4.2%	20.1%
$Ag_{0.2}Co_{0.8}/g\text{-}C_3N_4$	8.6%	19.2%
$Ag_{0.3}Co_{0.7}/g\text{-}C_3N_4$	14.2%	18.1%
$Ag_{0.7}Co_{0.3}/g\text{-}C_3N_4$	47.2%	11.1%
$Ag_{0.9}Co_{0.1}/g\text{-}C_3N_4$	58.9%	10.9%
$Ag_{1,0}Co_0/g\text{-}C_3N_4$	61.0%	0
